# Falciparum malaria and HIV-1 in hospitalized adults in Maputo, Mozambique: does HIV-infection obscure the malaria diagnosis?

**DOI:** 10.1186/1475-2875-7-252

**Published:** 2008-12-15

**Authors:** Aase Berg, Sam Patel, Nina Langeland, Bjorn Blomberg

**Affiliations:** 1 Stavanger University Hospital, Department of Medicine, Stavanger, Norway; 2 The Central Hospital of Maputo, Department of Medicine, Maputo, Mozambique; 3 The National Centre for Tropical Medicine, Department of Medicine, Haukeland University Hospital and Institute of Medicine, University of Bergen, Bergen, Norway

## Abstract

**Background:**

The potential impact of HIV-1 on falciparum malaria has been difficult to determine because of diagnostic problems and insufficient epidemiological data.

**Methods:**

In a prospective, cross-sectional study, clinical and laboratory data was registered consecutively for all adults admitted to a medical ward in the Central Hospital of Maputo, Mozambique, during two months from 28^th ^October 2006. Risk factors for fatal outcome were analysed. The impact of HIV on the accuracy of malaria diagnosis was assessed, comparing "Presumptive malaria", a diagnosis assigned by the ward clinicians based on fever and symptoms suggestive of malaria in the absence of signs of other infections, and "Verified malaria", a malaria diagnosis that was not rejected during retrospective review of all available data.

**Results:**

Among 333 included patients, fifteen percent (51/333) had "presumptive malaria", ten percent (28 of 285 tested persons) had positive malaria blood slides, while 69.1% (188/272) were HIV positive. Seven percent (n = 23) had "verified malaria", after the diagnosis was rejected in patients with neck stiffness or symptom duration longer than 2 weeks (n = 5) and persons with negative (n = 19) or unknown malaria blood slide (n = 4). Clinical stage of HIV infection (CDC), hypotension and hypoglycaemia was associated with fatal outcome. The "presumptive malaria" diagnosis was rejected more frequently in HIV positive (20/31) than in HIV negative patients (2/10, p = 0.023).

**Conclusion:**

The study suggests that the fraction of febrile illness attributable to malaria is lower in HIV positive adults. HIV testing should be considered early in evaluation of patients with suspected malaria.

## Background

The number of studies on prevalence and clinical manifestations of HIV 1 and falciparum malaria in Mozambique is limited, especially in adult inpatients. Mozambique is one of the countries in sub-Saharan Africa with the highest incidences of co-infection with HIV and malaria, with about 270 annual malaria cases per 1,000 inhabitants and an estimated HIV prevalence in adults of 16.1% in 2005 [1]. More than a quarter of the population in Maputo city may be HIV infected [2]. Nationally, approximately 3,500 die due to malaria annually [3]. Mozambique has stable malaria transmission with peak transmission from October – December to March-April [3], and *Plasmodium falciparum *accounts for 95% of the cases [4]. The quality of the data on HIV and malaria has been questioned in Mozambique and in several other developing countries [2,5-7].

There is controversy as to the degree of impact HIV has on malaria infection. Increasing severity of HIV is associated with increased risk of acquiring malaria infection, and of severe disease and death from malaria in areas with unstable malaria transmission [8-14]. Available studies are limited by low sample sizes and have failed to demonstrate any association between malaria case-fatality rates and HIV seropositivity among hospitalized patients in areas with stable malaria transmission [15]. Some postulate that HIV alters the clinical presentation of malaria [14]. Other investigators report increased treatment failure of antimalarials in HIV positive patients [16,17], whereas others contradict this finding [9,18].

Febrile illness is a common complaint in HIV patients and may be due to a multitude of other causes than malaria, including viral, bacterial and other parasitic or opportunistic infections, many of which may have clinical features indistinguishable from malaria [10,19]. Adverse effects of antiretrovirals and other medicines, including the immune reconstitution syndrome may also give rise to febrile illness [1]. The fact that people in endemic areas may have asymptomatic malaria parasitemia further complicates the diagnosis of febrile illness in HIV infected people [1,10,20].

The current study describes the prevalence and clinical manifestations of co-infection with HIV and malaria in hospitalized adult patients in Maputo and examines the impact of HIV on malaria infection with fatal outcome as primary end-point.

## Methods

### Setting

The Central Hospital of Maputo is a public tertiary teaching hospital that serves Maputo's 1.1 million citizens and also functions as national referral hospital for Mozambique. Patients are admitted on rotation, according to weekdays into the four medical wards.

### Study population

From the 28th October to the 28th December 2006, a prospective, cross-sectional study was carried out on all adult patients consecutively admitted to the medical ward no. 1 in the Central Hospital of Maputo. On admission and during daily ward rounds the ward medical doctors recorded observations in the patient's medical record. For patients with suspected infectious disease, a predefined set of clinical data was collected, including the duration of symptoms, the severity of the disease, the presence of clinical criteria for severe sepsis and severe malaria according to international definitions, clinical signs of HIV, the clinical stage of HIV disease according to the CDC (US Centers for Disease Control) and the degree of consciousness according to the Modified Glasgow Coma Scale. The recorded data included sex, age, and admission diagnosis, date of admission and discharge. All clinical information, as well as laboratory and x-ray results, was available for the researcher, who registered these into patient records during daily visits. Each patient record was observed on admission and followed up twice weekly, to observe alterations in the condition of the patient or in the diagnosis done. Most patients were also seen by the researcher together with the ward doctor who was responsible for the patient. Patients who were both admitted and discharged or who died during the same weekend, sometimes had limited observations and laboratory investigation performed. The data was crosschecked with the nurses' registries of deaths and HIV positive patients and with the ward statistic manager's registries of admissions, discharges, diagnoses and deaths. The patient outcome was dichotomized as either "discharged alive from the hospital" or "fatal outcome", meaning that the patient died during the hospital stay. Case-fatality rates were calculated as number of patients with "fatal outcome" divided by the total number of patients included in the study. When available, the results of HIV and malaria tests were recorded. Diagnoses on admission and discharge were registered. If there was a change in the clinical working diagnosis, the change was usually made during the first regular ward round after admission. All tests were performed on request from the clinicians. The HIV tests were usually requested during the first regular ward round. Pregnancy tests were not done routinely. Patients under 16 years of age were excluded from the study.

Two different definitions of malaria were used, "presumptive malaria" and "verified malaria". "Presumptive malaria" means that the clinicians in the ward have given the patient a working diagnosis of malaria based on symptoms suggestive of malaria such as fever, headache, mental confusion, vomiting and diarrhoea, difficulty in breathing, general body ache or other influenza-like symptoms, in the absence of symptoms or findings indicating other infections. Neck stiffness and history duration of more than two weeks were considered more consistent with other infections than malaria. The term "verified malaria" is used for patients, who have both clinical features of malaria ("presumptive malaria"), a positive malaria slide, and for whom the malaria diagnosis has not been rejected due to symptoms more consistent with other infections, such as neck stiffness or symptom duration of more than two weeks. Only patients with a documented positive HIV test were considered as HIV cases, and not those with clinically suspected HIV. Opportunistic infections were recorded when clinically suspected or when verified by tests.

### Laboratory methods

HIV tests were performed with both the Determine (Abbot Japan co, Ltd) and Unigold (Trinity Biotech plc, Bray, Ireland) tests. Malaria slides were prepared and evaluated as part of the hospital routine. Microscopy of Gram and Ziehl-Neelsen stained slides and culture for *Mycobacterium tuberculosis *were performed according to standard procedures in the hospital's microbiological laboratory.

### Statistical methods

Statistical analysis was performed with the Stata software (Stata Corps, College Station, TX). In the univariate analysis of risk factors for fatal outcome, odds ratios, 95% confidence intervals and p-values were calculated. In the multivariable analysis of risk factors for fatal outcome, all explanatory variables with a univariate significance level of p < 0.2 were included in the initial model. Automated stepwise backwards logistic regression modelling was performed, removing variables with p-values < 0.2 in order of decreasing p-values. HIV infection was included both as a dichotomized variable (infected or not infected) and as the degree of immunosuppression recorded as an ordinal variable with the values "0" for not infected and "1" through "3" for those with HIV infection with CDC (US Centers for Diseases Control) clinical stage from A through C, respectively. The *a priori *important variables, age (number of years) and sex were included in the model to adjust for potential confounding, but removed from the model on equal terms as other variables when p-values exceeded 0.2. For the purpose of analysis of risk factors for fatal outcome, an assumption was made for the variables "neck stiffness", "diarrhoea", "cough" and "hypotension" that a missing value equalled absence of the particular feature. Likewise, for the tests of serum creatinine, platelet counts and blood sugar, missing values were grouped together with normal test results for the purpose of risk factor analysis. Assessment of the association between HIV status and malaria was performed by Fisher's exact test. The association between HIV status and the risk of receiving a wrong malaria diagnosis was evaluated by Fisher's exact test, and confounding by age and sex was evaluated by logistic regression. Risk of fatal outcome among patients with verified and rejected final diagnosis of malaria was evaluated by Fisher's exact test.

### Ethical considerations

The data recorded were obtained as part of routine clinical examination and routine blood tests. No interventions were done and no additional testing was initialized on behalf of the study. Patients were recorded anonymously, without names or other traceable identification. To prevent mixing of patients, admission dates and bed-numbers, which together were unique for each and every patient, were recorded. The National Ethical Committee at the Ministry of Health in Mozambique approved the study and granted permission that the study could be performed without asking for the consent from the patients.

## Results

### Characteristics of the study population

A total of 333 adult patients were included in the study, and among these 272 patients (81,7%) were tested for HIV, 285 (85.6%) were tested for malaria and 241 (72.4%) had both tests done. The median age was 38 years (range 16–92). The median age for HIV positive patients, HIV negative patients and for patients with verified malaria was 33, 49 and 34 years, respectively. The proportion of females was 50% in the total study population, 51% in the HIV positive patients and 39% in the patients with verified malaria.

### Prevalence of HIV and malaria

Among those tested, sixty-nine percent (188/272) had a positive HIV test. Fifteen percent (51/333) of the study subjects were diagnosed with "presumptive malaria" on clinical grounds. Malaria parasitemia was found in ten percent (28/285) of those tested. After retrospective review of all information available for the 28 patients with positive malaria blood slide, the malaria diagnosis was discarded for five patients who had neck stiffness or duration of symptoms over two weeks, considered clinical signs more consistent with other infections than malaria, the most common being meningoencephalitis and tuberculosis. Thus, in retrospect, seven percent (23/333) of the total study population were given a final diagnosis of "verified malaria". Among the 23 patients with "verified malaria", eleven were HIV positive. All five patients with malaria parasitemia who had their malaria diagnosis rejected were HIV positive. Ninety-two patients had neither a HIV test nor a malaria blood slide done.

### Case-fatality rates

The in-hospital case-fatality rate was 15.3% (51/333) for the total study population. Having a positive HIV test was not associated with a significantly higher case-fatality rate. However, trend analysis showed that the severity of HIV disease was significantly associated with increasing case-fatality rates (CDC stage A: 4.5% (1/22), stage B: 16.7% (5/30) and stage C: 21.0%, (30/143), odds ratio 1.36 (1.04–1,78), p = 0.026). Neither a positive malaria test nor increasing malaria parasitemia was significantly associated with fatal outcome (table 1). Neither a diagnosis of "presumptive malaria" nor "verified malaria" was associated with an increased risk of fatal outcome. Two died among the five malaria parasitemic patients, who had the malaria diagnosis rejected because clinical features were more consistent with other diagnoses. However, there was no statistically significant difference in the case-fatality rates between patients with "verified malaria" (8.7%, 2/23) and those with a retrospectively rejected malaria diagnosis (21.4%, 6/28, OR: 2.86 (95%CI 0.52–15.81) p = 0.227). A total of 92 patients did not have known test results for HIV (n = 44), malaria (n = 31) or both HIV and malaria (n = 17). There was no statistically significant case-fatality rate between the 92 patients with missing data for HIV and/or malaria (13.0% 12/92) and the 241 patients who had known HIV and malaria test results (16.2% 39/241, OR: 1.29 (95%CI: 0.64–2.59) p = 0.478). Increasing age was not associated with increased risk of fatal outcome (Table 1).

**Table 1:** Risk factors for fatal outcome

**Characteristic**	Case fatality rate (deaths/no at risk)*	**Univariate analysis OR (95%CI)**	**p**	**Multivariate analysis, adjusted OR (95%CI), P †**
Overall	15.3% (51/333)			
Age (no of years)‡	NA	0.98 (0.97–1.00)	0.071	Removed (p = 0.433)
Sex:			0.682	Removed (p = 0.257)
Female	14.5% (24/165)	1		
Male	16.2% (27/167)	1.13 (0.62–2.06)		
Malaria parasitemia			0.859	
Negative	15.6% (40/257)	1		
Positive	14.3% (4/28)	0.90 (0.30–2.75)		
Malaria parasitemia (graded 1–5)‡	NA	1.02 (0.75–1.40)	0.889	
High parasitemia			0.261	
<5% of erythrocytes	14.8% (40/270)	1		
≥ 5% of erythrocytes	27.3% (3/11)	2.16 (0.55–8.52)		
Initial diagnosis			0.936	
Other than malaria	15.3% (43/282)	1		
Presumptive malaria	15.7% (8/51)	1.03 (0.45 – 2.35)		
Retrospective diagnosis			0.166	Removed (p = 0.401)
Other than malaria	20.9% (39/187)	1		
Verified malaria	8.7% (2/23)	0.36 (0.08 – 1.62)		
HIV test result			0.103	Removed (p = 0.290)
Negative	10.7% (9/84)	1		
Positive	18.6% (35/188)	1.91 (0.87–4.19)		
CDC HIV stage‡§	NA	1.36 (1.04–1.78)	0.026	1.87 (1.01–3.47) p = 0.048
Anti retroviral therapy			0.953	
Not on ART	15.4% (45/293)	1		
On ART	15.0% (6/40)	0.97 (0.39–2.45)		
Hypotension			0.057	4.02 (1.01–15.89) p = 0.048
No hypotension reported	14.5% (46/318)	1		
Blood pressure <90 mmHg	33.3% (5/15)	2.96 (0.97–9.04)		
Neck stiffness			0.323	
No neck stiffness reported	15.8% (50/317)	1		
Stiff neck	6.3% (1/16)	0.36 (0.05–2.76)		
Cough			0.757	
No cough reported	14.9% (36/241)	1		
Coughing	16.3% (15/92)	1.11 (0.58–2.14)		
Diarrhoea			0.862	
No diarrhoea reported	15.5% (43/278)	1		
Diarrhoea present	14.5% (8/55)	0.93 (0.41–2.11)		
Serum creatinine			0.907	
< 175 μmol/L or not tested	15.2% (44/289)	1		
≥ 175 μmol/L	15.9% (7/44)	1.05 (0.44–2.51)		
Platelet counts			0.008	2.23 (0.94–5.33) p = 0.070
≥ 100 × 10^9^/L or not tested	13.1% (37/283)	1		
<100 × 10^9^/L	28.0% (14/50)	2.59 (1.27–4.5.25)		
Blood-glucose			<0.001	13.57 (2.69–68.45) p = 0.002
≥ 2.2 mmol/L or not tested	14.2% (46/324)	1		
<2.2 mmol/L	55.6% (5/9)	7.55 (1.96–29.18)		

CFR case-fatality rate, OR odds ratio, 95%CI 95% confidence interval, NA not applicable, CDC Centers for Disease Control, MPS = malaria parasite slide. * The number of observations in the CFR column varies because some patients' test results were not known. † 189 observations in final model ‡The Odds Ratio estimates for quantitative/ordinal variables are approximations to the odds ratio for a one unit increase. § 0 = negative, 1 = CDC stage A, 2 = CDC stage B, 3 = CDC stage C.

### Clinical manifestations

Although the most serious cases were transferred to the intensive care unit, there was a high frequency of features indicating severe illness in the study population. In the comparison of symptoms, findings and laboratory results of patients with final "verified malaria" diagnosis and those with a final rejected malaria diagnosis, headache was the only feature significantly associated with a final diagnosis of "verified malaria" (34.8%, 8/23) as compared to those with a final "rejected malaria" diagnosis (3.6%, 1/28, OR: 14.4 (95%CI: 1.59–661.85) p = 0.004). There was no significant difference in symptoms, clinical findings and laboratory results between the HIV positive and the HIV negative malaria patients (Table 2).

**Table 2:** The clinical manifestations of patients with presumptive malaria according to HIV status

	**Verified malaria**			**Rejected malaria diagnosis**			**All**
	**HIV+**	**HIV-**	Total*	**HIV+**	**HIV-**	Total*	
Total, n	12	8	23	20	2	28	51
Median age, years (n)	30 (11)	39 (8)	34 (23)	32 (20)	52 (2)	32 (28)	34 (51)
Female sex, n (%)	5 (42)	4 (50)	9 (39)	10 (50)	0	12 (43)	21 (41)
Median symptom duration, days (n)	7 (8)	3 (7)		7 (17)	7 (1)		
	n (%)	n (%)	n (%)	n (%)	n (%)	n (%)	n (%)
							
Vomiting	5 (42)	7 (88)	12 (52)	12 (60)	0 (0)	13 (46)	25 (49)
Diarrhoea	5 (42)	0 (0)	5 (22)	5 (25)	0 (0)	7 (25)	12 (24)
Headache †	3 (25)	4 (50)	8 (35)	0 (0)	1 (50)	1 (4)	9 (18)
Arthralgia	1 (8)	3 (38)	5 (22)	0 (0)	0 (0)	0 (0)	5 (10)
Dyspnoea	0 (0)	0 (0)	0 (0)	0 (0)	1 (50)	1 (4)	1 (2)
Loss of weight	1 (8)	0 (0)	0 (0)	5 (25)	0 (0)	5 (18)	6 (12)
Abdominal pain	1 (8)	1 (13)	2 (9)	0 (0)	0 (0)	0 (0)	2 (4)
Cough	1 (8)	0 (0)	2 (9)	8 (40)	0 (0)	8 (18)	7 (14)
Convulsions	0 (0)	0 (0)	0 (0)	2 (10)	1 (50)	4 (14)	4 (8)
Fever>4 weeks	0 (0)	0 (0)	0 (0)	2 (10)	0 (0)	2 (7)	2 (4)
							
Temperature>38.5°C	7 (58)	6 (75)	13 (57)	10 (50)	2	15 (54)	28 (55)
BP < 90 mmHg	0 (0)	0 (0)	1 (4)	4 (20)	1 (50)	6 (21)	7 (14)
Heart rate >100/min	3 (25)	1 (13)	5 (22)	10 (50)	0 (0)	10 (36)	15 (29)
Respiratory rate >20/min	4 (33)	0 (0)	5 (22)	8 (40)	1 (50)	9 (32)	14 (27)
Jaundice	2 (17)	1 (13)	3 (13)	2 (10)	0 (0)	2 (7)	5 (10)
Mental confusion	4 (33)	1 (13)	5 (22)	4 (20)	1 (50)	8 (29)	13 (25)
Cachexia	1 (8)	0 (0)	1 (4)	2 (10)	0 (0)	2 (7)	3 (6)
Neck stiffness	0 (0)	0 (0)	0 (0)	7 (35)	0 (0)	7 (25)	7 (14)
Hepatomegaly	0 (0)	0 (0)	0 (0)	1 (5)	0 (0)	1 (4)	1 (2)
Splenomegaly	0 (0)	0 (0)	0 (0)	1 (5)	0 (0)	1 (4)	1 (2)
WBC<4 or >12 × 10^9^/L	3 (25)	1 (13)	5 (22)	7 (35)	1 (50)	9 (32)	14 (27)
Platelets<100 × 10^9^/L	6 (50)	5 (63)	13 (57)	6 (30)	1 (50)	8 (29)	21 (41)
Creatinine>115 μmol/L	6 (50)	2 (25)	9 (39)	11 (55)	1 (50)	13 (46)	22 (43)
Blood glucose<2.2 mmol/L	0 (0)	0 (0)	0 (0)	2 (10)	1 (50)	3 (11)	3 (6)
HIV Stage A	0 (0)	NA	0 (0)	0 (0)	NA	0 (0)	0 (0)
HIV Stage B	2 (17)	NA	2 (9)	1 (5)	NA	1 (4)	3 (6)
HIV Stage C	7 (58)	NA	7 (30)	11 (55)	NA	11 (39)	18 (35)
MPS positive	12 (100)	8 (100)	23 (100)	5 (25)	0 (0)	5 (18)	28 (55)
Fatal outcome	1 (8)	1 (13)	2 (7)	4 (20)	0 (0)	5 (18)	7 (14)

BP systolic arterial blood pressure, WBC white blood cells. *Some of the totals don't add up, as some patients with unknown HIV result were included in the total, but not in the rest of the table. † Headache was significantly associated with a "verified malaria" diagnosis (p = 0.007)

### HIV-malaria interaction

The proportion of patients with malaria parasitemia was not significantly different between HIV positive (9.8%, 16/164) and HIV negative (10.4%, 8/77, OR: 0.93 (95%CI: 0.36–2.64) p = 0.878). In patients with documented malaria parasitemia, the degree of parasitemia (graded from 1 to 5) was not significantly different between HIV-positive (mean 2.8, 95%CI: 1.9–3.7) and HIV-negative patients (mean: 3.5, 2.2–4.8, p = 0.170). In five patients with documented malaria parasitemia, the malaria diagnosis was rejected because other more plausible causes of the symptoms were found, and all of these five were HIV positive. Still, the difference in this assumed coincidental, not clinically relevant malaria parasitemia was not statistically significant between HIV positive (31.3%, 5/16) and HIV negative patients (0%, 0/8, p = 0.130). Upon retrospective analysis of the data, the malaria diagnosis was rejected in more than half (n = 28) of the 51 patients, who were given a working diagnosis of "presumptive malaria" while in the ward. The malaria diagnosis was rejected significantly more frequently in HIV positive (64.5%, 20/31) than in HIV negative patients (20%, 2/10, OR: 7.27 (95%CI: 1.31–40.42) p = 0.023). The malaria diagnosis was also rejected in 6 of 10 patients with unknown HIV status. As shown in Table 3, HIV remained a risk factor for having a rejected malaria diagnosis, even after adjusting for sex and age. There was a borderline, but not statistically significant association between increasing severity of immunosuppression and a rejected malaria diagnosis (OR: 1.69, 95%CI: 0.99–2.89, p = 0.54).

**Table 3:** Multivariate analysis of risk factors for receiving an incorrect diagnosis of malaria (number of observations = 40)

Characteristic	Adjusted OR	95%CI	p
Male sex	1.03	0.26 – 4.10	0.969
Age *	1.01	0.96 – 1.07	0.626
HIV infection	9.29	1.45 – 59.62	0.019

OR odds ratio, 95%CI 95% confidence interval. *Age is included as a continuous variable with number of years.

The case-fatality rate for patients with a rejected malaria diagnosis was 21.4% (6/28) and for those with a verified malaria diagnosis it was 8.7% (2/23), however, this difference was not statistically significant (OR: 2.86 (95%CI: 0.50–16.51), p = 0.269). Among patients with a diagnosis of "verified malaria" there was no significant difference in case-fatality rates among those who were HIV-positive (9.1%, 1/11) and HIV-negative (12.5%, 1/8, OR: 0.70 (95%CI: 0.03–14.35) p = 1.0). Among HIV-positive patients, only one person with verified malaria died (9%, 1/11), while four persons with rejected malaria diagnosis (20%, 4/20) died, but this difference was not statistically different (OR: 2.50 (95%CI: 0.23–27.34) p = 0.631).

## Discussion

This study is one of a few reports addressing the issue of malaria and HIV co-infection in Mozambique. In the study, risk factors for fatal outcome were hypoglycaemia, hypotension and severity of immunosuppression. Malaria parasitemia and degree of parasitemia was not significantly associated with fatal outcome. Upon retrospective review of all available data, the malaria diagnosis was rejected in more than half of the patients who initially had received a working diagnosis of "presumptive malaria" while in the ward. HIV positive patients were significantly more likely to have their malaria diagnosis rejected than HIV negative ones. Thus, the fraction of febrile illness attributable to malaria in this study is less in HIV positive than in HIV negative persons. The finding probably reflects that HIV positive patients are prone to a number of additional opportunistic infections and febrile illnesses which may be difficult to distinguish clinically from malaria, and thus, they run a greater risk of being misdiagnosed as having malaria. Other investigators have raised concern regarding the reliability of malaria diagnosis in HIV patients [10,19,20]. Our study demonstrates a statistically significant association between HIV status and a risk of receiving an incorrect malaria diagnosis. The findings raise concern that current practice may lead to overuse of antimalarials and may leave a number of HIV patients with opportunistic infections undiagnosed and untreated. However, as the study population is small, further studies should be performed to clarify this issue.

The difficulties in diagnosing malaria in HIV positive individuals are, at least partly, due to the fact that a number of opportunistic and other infections in HIV patients may mimic malaria. Even in HIV negative patients, malaria can be clinically indistinguishable from other infections such as viral infections or septicaemia [21,22]. The fact that a proportion of people in endemic areas have asymptomatic malaria parasitemia increases the diagnostic challenges [23,24]. Adverse effects of antiretrovirals and immune reconstitution syndrome further complicate the diagnostic process [1].

Clinicians in low-resource settings often face diagnostic challenges due to a multitude of factors, including high workload, poor facilities, unavailability and underuse of equipment and laboratory tests and lack of access to up-dated scientific literature and continued post-graduate education programs [5,25-27]. The findings of this study emphasize that the diagnosis of malaria is less straight-forward in HIV patients than in HIV negative ones, and that it is important to consider differential diagnoses, particularly opportunistic infections, in febrile HIV infected persons living in malaria endemic areas. In this study, meningoencephalitis and tuberculosis were the most common diagnoses that were mistaken for malaria. Febrile patients with suspected or confirmed HIV infection, particularly those, who have neck stiffness or duration of symptoms longer than two weeks, should be carefully evaluated for differential diagnoses other than malaria. The possibility of a double infection with malaria in addition to another infection must also be considered.

Neither this study, nor a study from Burkina Faso [15] demonstrated any increased risk of severe malaria infection or fatal outcome of malaria in HIV positive patients, but the small sample sizes in both studies may limit the studies' ability to evaluate that question. However, in areas with unstable malaria transmission, studies have shown an association between HIV-status and severity and lethality of malaria [14,27]. A potential bias in several of the studies on malaria severity and mortality in HIV patients may be the pre-selection or inclusion of only patients with relatively mild malaria infection [8,10,20,28]. While previous studies on the outcome of malaria largely used other treatment regimens [9,12,16], most severe malaria cases in the current study were treated with the highly efficient artesunate combination therapy, which may have reduced the case-fatality rates. Since other studies have indicated that acquired immunity against malaria is important when there is partial drug-resistance [29], the use of artesunate combination therapy may diminish any differences in treatment success between HIV positive and HIV negative individuals.

The low prevalence of malaria parasitemic patients may be a result of the increased government eradication effort in the main patient recruiting area, but may also be a consequence of false negative malaria tests due to intake of effective or ineffective antimalarials prior to admission, or inaccurate laboratory diagnosis. Using thick malaria blood smears only in the malaria diagnosis may result in an increased number of false negative blood slides [25]. Since malaria in HIV positive patients is known to present with uncharacteristic clinical features, HIV positive patients and those with unknown HIV status whom were not tested for malaria, may represent a potential bias underestimating the prevalence of malaria. However, the case-fatality rate in the group with unknown results for HIV and/or malaria was not significantly different from those with known results.

Hypoglycaemia was associated with increased risk of fatal outcome and the only clinical finding associated with a rejected malaria diagnosis. Hypoglycaemia may be an adverse effect of quinine treatment, which 11% of the patients received, or other medicines such as trimethoprim-sulfamethoxazole, which 44% received. Alternatively, the hypoglycaemia may be due to malnutrition, other super-infection, a double infection with both malaria and another infection, or an independent indicator of severe disease [30].

Potential biases of the study include possible underreporting of the HIV and malaria, since malaria and/or HIV test was not performed for 92 patients. Particularly, since HIV-patients may present with atypical signs and symptoms of malaria [1,14], there is a possibility that HIV infected study subjects, with known or unknown HIV status, may have suffered from undiagnosed malaria. Patients with low or no incomes may be underrepresented due to the admission fee of approximately 6 USD for those who do not have a referral letter, while wealthy patients may be underrepresented because they tend to prefer private health care. This may represent a bias, since lower socio-economical status is associated with poorer housing and lower use of impregnated bed nets, and hence higher frequency of malaria [31]. Furthermore, relatively healthy patients were treated as outpatients and not admitted while some severely ill patients were transferred to the intensive care unit. The severity of disease in this cohort reflects that patients, screening nurses at the primary health care level and doctors in the Emergency Unit have a high threshold for referring and admitting patients to the hospital. Among all patients attending the Emergency Unit with suspected malaria infection during one week, about half of the patients were discharged directly after some hours of observation. Patients admitted during weekends, who either were discharged or died before the next working day, probably represent the least ill and the most severely ill patients, respectively, and since there was less clinical follow-up and laboratory investigation performed during weekends, these patient groups may represent a selection bias. Finally, pregnancy tests were not routinely done in this ward, and thus, hidden pregnancies may represent a bias giving increased severity of the malaria infection. However, no difference in severity of malaria between the sexes was observed.

## Conclusion

The severity of immunosuppression due to HIV-stage, hypoglycaemia and hypotension were associated with fatal outcome in this study. The findings of the study indicate that the fraction of febrile illness attributable to malaria is less in HIV positive than in HIV negative persons. This finding is plausible since opportunistic and other infections may mimic malaria in HIV patients. It raises concern of overuse of antimalarials and missed treatment opportunities for opportunistic infections in HIV positive patients. In patients presenting with febrile illness in areas where both HIV and malaria are endemic, clinicians must make additional efforts to rule out opportunistic infections and other differential diagnoses to malaria. Knowledge of HIV status is valuable in the diagnostic evaluation of patients with suspected malaria, and HIV testing should be considered as part of the early evaluation.

## Authors' contributions

AaB planned the study, collected the data, carried out the analysis and interpretation of data and prepared the manuscript. SP contributed in the planning of the study, the data collection and in the preparation of the manuscript. NL contributed in the planning of the study, analysis and interpretation of the data and in the preparation of the manuscript. BB contributed in the planning of the study, carried out the analysis and interpretation of the data and contributed to the preparation of the manuscript.
